# Breast cancer screening attitudes, beliefs, and behaviors of Zuni Pueblo women: identifying cornerstones for building effective mammogram screening intervention programs

**DOI:** 10.1007/s10552-023-01814-8

**Published:** 2023-11-09

**Authors:** Kate Cartwright, Deborah Kanda, Mikaela Kosich, Judith Sheche, Samantha Leekity, Nicholas Edwardson, V. Shane Pankratz, Shiraz I. Mishra

**Affiliations:** 1 School of Public Administration, University of New Mexico, Albuquerque, NM USA; 2 Comprehensive Cancer Center, University of New Mexico, Albuquerque, NM USA; 3 Department of Internal Medicine, University of New Mexico, Albuquerque, NM USA; 4 Departments of Pediatric and Family and Community Medicine, University of New Mexico, Albuquerque, NM USA

**Keywords:** Breast cancer screening, American Indian/Alaska Native health, Health equity

## Abstract

**Purpose:**

Breast cancer is the leading form of cancer and has the second highest mortality rate of cancers for American Indian/Alaska Native (AI/AN) women. Early screening is critical. This study examines the breast cancer-related knowledge, beliefs, and behaviors of Zuni women in the Southwest United States (U.S.).

**Methods:**

In 2020 and 2021, a survey was administered to better understand cancer screening patterns in Zuni Pueblo; 110 women from 50 to 75 years of age were recruited to respond to the breast cancer screening portion. Inclusion criteria included self-identifying as AI, a member of the Zuni tribe, or married to a Zuni tribal member, and meeting the age and gender requirements. Descriptive statistics and bivariate analyses were conducted examining the associations between measures of breast cancer knowledge, beliefs, and behaviors and breast cancer screening status (never, ever/non-compliant, and ever/compliant).

**Results:**

Of survey participants, 47.3% have had a breast cancer screening and are up-to-date, 39.1% have had a screening in the past but are not up-to-date, and 13.6% have never been screened. Age was the only statistically significant socioeconomic predictor of breast cancer screening; the median (interquartile range) ages of each group are 62 (54, 68) ever/compliant, 56 (54, 68) ever/non-compliant, and 53 (51, 55) never (*p*-value < 0.001). Significant differences by health status and access to medical care include having a regular health care provider and going to see a provider for routine check-ups. The survey also shows differences in knowledge about breast cancer risk factors, beliefs, and behaviors. Women across all three screening statuses reported that they would get screened if encouraged by a health care provider.

**Conclusion:**

While survey respondents report a relatively high rate of ever having had a breast cancer screening, less than half are compliant with screening guidelines, which shows there is an opportunity to improve breast cancer screening rates. With culturally tailored interventions, providers have the potential to improve breast cancer screening for Zuni women.

## Introduction

Similar to the wider population of women in the US, breast cancer is the leading form of cancer for American Indian/Alaska Native (AI/AN) women. As of 2019, breast cancer is the top cancer by the rate of new cancer cases for AI/AN women (73.1 cases/100,000 women) and is the second highest cancer leading to mortality for AI/AN women [1]. AI/AN women tend to have lower rates of breast cancer and breast cancer mortality compared to non-Hispanic white women [2]. From 2014 to 2018, the breast cancer incidence rate per 100,000 for AI/AN women was 81.7 compared to 137.9 for non-Hispanic white women (a ratio of 0.6) and the breast cancer mortality rate per 100,000 for AI/AN women was 14.8 compared to 20.1 for non-Hispanic white women (a ratio of 0.7) [2]. However, these lower incidence patterns do not minimize the burden of breast cancer for AI/AN communities where breast cancer is still among the most common malignancy and continues to lead to considerable morbidity and mortality [2]. Additionally, tracking statistics over time, from 1999 to 2015, AI/AN women are experiencing a relative increase in new cases of breast cancer compared to non-Hispanic white women [3].

One of the most impactful ways of minimizing the burden of breast cancer is to detect it at the earliest possible stage through guidelines compliant screening, specifically mammography. There is wide variation in information about AI/AN mammogram patterns based on the source of the data. Self-reported data from the 2019 National Health Interview Survey, indicate that AI/AN women and non-Hispanic white women have similar rates of mammography screening: 65.3% of AI/AN women compared to 68.0% of non-Hispanic white women [2]. However, drawing from clinical data from the national Indian Health Service (IHS) performance statistics, the percentage of AI/AN women aged 52–74 who have received mammography screening from the IHS within the previous 2 years is 42.6 in 2018 and 42.0 in 2019. Of the Albuquerque Area IHS population, 43.0% (2018) and 38.7% (2019) of women have received mammography screening within the last 2 years [4]. These clinical figures may point to screening inequities. Additionally, AI/AN breast cancer screening patterns vary based on Tribe and region. While these discrepancies may be partially due to self-report versus clinical data and to some individuals seeking and receiving care outside of the IHS, all sets of figures demonstrate that AI/AN women breast cancer screening percentages fall short of the Healthy People 2020 goal for breast cancer screening of 81.1% [4]. Early screening is critical for minimizing the burden of breast cancer for AI/AN women.

To improve breast cancer screening in AI/AN women, studies need to prioritize understanding motivators for and barriers to screening. Access to timely and regular breast cancer screening is key to minimizing the burden of breast cancer and minimizing related health disparities [5]. Studies show that racial and ethnic minority populations including AI/AN have specific factors which both facilitate and hamper breast cancer screening [6–9]. Prior studies have demonstrated an association between breast cancer screening awareness and breast cancer screening behaviors [9]. Studies exploring factors hindering AI/AN breast cancer screening connect lack of knowledge about breast cancer screening and risks, in addition to sociodemographic factors, to lower rates of breast cancer screening. AI/AN women report culturally specific views and beliefs about breast cancer screening like mammography [7, 10].

An additional factor that has been identified as a facilitator and a detractor at times is the IHS provision of services [11]. The IHS provides more accessible care in many ways, but also due to systematic underfunding from the federal government in addition to other factors, the IHS may also prevent or delay access to breast cancer screening services for AI/AN women. AI/AN populations are entitled by treaty to receive healthcare free of charge provided by the US federal government, which for most is healthcare provided by the Indian Health Service (IHS). This universal health care coverage does not result in easily accessible health care services in many cases [12]. Challenges to health care access through the IHS stem from several factors including underfunding, geographic sparsity, and historic trauma leading to current mistrust [12]. While AI/AN populations are entitled to healthcare, the federal funding is insufficient for addressing comprehensive healthcare needs. In 2019, the annual IHS expenditure per user population was $4,078 compared to the national average of over $10,000 per person [13]. An additional challenge for the AI/AN population in accessing care is geography. While there are over 300 IHS primary health clinics, there are only 46 IHS hospitals, and not all hospitals provide specialty care services [12]. This means that in many cases AI/AN patients and caretakers must travel long distances to receive specialty healthcare services, such as mammograms. Finally, the HIS has a complicated history of healthcare delivery, which included culturally destructive healthcare programs, such as a program in the 1970s which resulted in the sterilization of over 25% of AI/AN women of childbearing age without adequate consent [14]. While the current leadership and practices and policies aim to center patient autonomy and many efforts have been made to build strong, positive relationships between IHS facilities and Tribal communities, many AI/AN women are justifiably wary of receiving IHS services due to historic trauma [14]. Engagement with IHS should be further explored to better understand the link between cancer-specific care and reducing cancer disparities [11].

Ultimately, the literature shows that breast cancer studies are not racially, ethnically, nor geographically representative. There is a need for more breast cancer and breast cancer screening research focused on AI/AN women. AI/AN experiences with cancer screening are not uniform; there is a need for more Tribe-specific research. There is great diversity among the 574 federally recognized Tribes, yet the vast majority of research investigates AI/AN health patterns as one group and generalizes findings based on these studies [10, 15]. This project answers that call for Tribe-specific research and addresses priority goals for Zuni Pueblo to improve cancer outcomes; recent research demonstrates that Zuni Pueblo has unique resources, strengths, and challenges in addressing cancer inequities [15–19]. This study contributes to this growing scholarship by examining the breast cancer-related knowledge, beliefs, and behaviors of Zuni women in the Southwest. Finding from this study will inform the development, implementation, and resting of interventions tailored to the unique attributes of Zuni women.

## Materials and methods

### Research setting

In 2020 and 2021, a survey was administered in partnership with the Zuni Health Initiative in the Zuni Pueblo, the largest Pueblo Tribe in New Mexico with approximately 11,000 residents, to better understand cancer screening patterns for cervical, breast, and colorectal cancers. While Zuni is a small, rural community, breast cancer screening including mammography is available locally at the IHS-administered Zuni Comprehensive Health Center [20]. This project has received research approval from the Zuni Pueblo Tribal Council, the Southwest Tribal Institutional Review Board (IRB), and UNM Health Sciences Center IRB.

### Sampling strategy and eligibility criteria

The sampling strategy for this community survey includes random sampling of streets, strategic convenience sampling, and snowball sampling. This survey sample is the result of inclusive, broad reaching recruitment efforts; however, this survey is not a systematic, representative random sample. The sampling strategy began with a random selection from all streets in Zuni Pueblo after which all residences located on selected streets were recruited through flyers. Women called the number listed on the flyer and the research team also conducted limited in-person outreach efforts. Research team members would inform potential participants about the study when they went to households to distribute the fliers. COVID-19 restrictions limited most in-person recruitment strategies, but recruiting efforts were supplemented through outreach at high-traffic community locations and snowball sampling from key community stakeholders and respondents. Inclusion criteria included self-identifying as AI, a member of the Zuni tribe, or married to a Zuni tribal member, and meeting the age and gender requirements for the age/gender-specific survey. To be eligible for the breast cancer screening portion of the survey, in addition to the above criteria, women needed to be at least 50 and up to 75 years of age, resulting in 110 respondents.

### Study implementation

The survey used an observational, cross-sectional design. The Zuni Health Initiative (ZHI) staff conducted surveys between October 2020 and April 2021. The survey varied in length based on the age/gender-specific survey. The survey varied by three groups: women 21–49, women 50–75, and men 50–75. All groups were asked the same questions about general cancer knowledge, beliefs, and behaviors as well as demographics. Women 21–49 were asked the cervical cancer knowledge, beliefs, and behaviors subset; women 50–75 were asked the cervical, breast, and colorectal cancer knowledge, beliefs, and behaviors subset; and men 50–75 were asked the colorectal cancer knowledge, beliefs, and behaviors subset. All participants received a merchandise card in recognition of their participation in the study. Due to COVID-19 pandemic precautions, surveys were conducted by phone.

### Measures/variables

*Dependent variable* Breast cancer screening status is determined by self-report of mammograms and the time of last mammogram at the time of the survey based on the breast cancer screening recommendations from the US Preventive Services Task Force (USPSTF): ever screened/compliant includes women who have been screened within the last 2 years; ever screened/non-compliant includes women whose last screening was over 2 years ago; and never screened includes women who have never had a mammogram [21].

*Independent variables* Variables of interest include a wide range of factors associated with cancer screening and cancer outcomes. *Sociodemographic* characteristics include age, marital status, language, education, income, and employment. In addition to mammogram status and most recent year of mammogram, *breast cancer screening behaviors* of interest include the main reason for having most recent mammogram, reasons for not having had a mammogram, and questions about when women should start having mammograms. These questions are adapted from the measures included in the National Cancer Institute Health Information National Trends Survey (HINTS) [22]. *Health status and access to medical care* measures include self-rated health, personal and family history of cancer, and multiple questions about personal experiences with healthcare. *Breast cancer risk knowledge* is measured by asking women to select which of 21 factors (10 more medically reasonable, and 11 less medically reasonable) increase a woman’s chance of developing breast cancer. These questions were compiled from the risk factors listed by the American Cancer Society, the National Cancer Institute, and the Centers for Disease Control and Prevention and are listed in the results tables [23–26]. These factors have been used in previous research and were updated with the current best evidence [27, 28]. *Attitudes and beliefs about cancer in general* include general statements about cancer (agree/disagree), measures social network attitudes and beliefs, attitudes and beliefs about healthcare providers and cancer screening, and future screening intentions. Finally, women reported *preferences for strategies to improve cancer screening*, which include 15 evidence-based strategies designed to increase cancer screening from the Community Preventive Services Task Force’s Community Guide (based off recommendations for breast, cervical, and colorectal cancers) [29].

### Data analysis

Descriptive statistics were calculated by mammogram screening status for sociodemographic characteristics, health status and access to medical care, breast cancer risk knowledge, breast cancer screening behaviors, attitudes and beliefs toward cancer in general, and preferences for strategies to improve breast cancer screening. Bivariate assessments of significant differences among the groups were accomplished using the statistical approach appropriate for the data type (i.e., Fisher’s exact, Pearson’s Chi-squared, or Kruskal–Wallis rank sum tests).

## Results

*Sociodemographic characteristics* Of Zuni women who participated in the survey, 47.3% have had a breast cancer screening and are compliant, 39.1% have had a screening in the past but are non-compliant, and 13.6% have never been screened. There were few meaningful and statistically significant characteristics associated with breast cancer screening status (Table 1). Age was the only meaningful and statistically significant socioeconomic covariate associated with breast cancer screening; the median [interquartile range (IQR)] ages of each group are 62 (54, 68) years for the ever/compliant, 56 (54, 68) years for the ever/non-compliant, and 53 (51, 55) for the never (*p*-value < 0.001). Employment status was also significantly different among the groups (*p*-value 0.027). Noteworthy patterns were identified among non-statistically significant associations: in all breast cancer screening groups, the vast majority speak a language other than English at home (96% ever/compliant, 86% ever/non-compliant, and 93% never) and at least 80% of respondents report having health insurance across all groups (86% for both ever/compliant and ever/non-compliant and 80% never).

**Table 1:** Sociodemographic characteristics of participants by mammogram screening status (*n* = 110)

	Compliant (*n* = 52)	Ever had test but not compliant, (*n* = 43)	Never had test (*n* = 15)	*p* value
Age	62 (54, 68)	56 (54, 68)	53 (51, 55)	**< 0.001**
BMI	29 (25, 32)	29 (26, 32)	27 (25, 32)	0.7
Married/partnered				0.7
No	26 (50%)	24 (56%)	9 (60%)	
Yes	26 (50%)	19 (44%)	6 (40%)	
Speak non-English at home?				0.2
No	2 (3.8%)	6 (14%)	1 (6.7%)	
Yes	50 (96%)	37 (86%)	14 (93%)	
Fluency speaking English				0.13
English only	2 (3.8%)	6 (14%)	1 (6.7%)	
Very well	22 (42%)	23 (53%)	9 (60%)	
Well	24 (46%)	14 (33%)	4 (27%)	
Not well	4 (7.7%)	0 (0%)	1 (6.7%)	
Education				0.4
High school or less	29 (56%)	18 (42%)	8 (53%)	
More than high school	23 (44%)	25 (58%)	7 (47%)	
Income				0.3
< $10,000	18 (35%)	15 (36%)	7 (47%)	
$10,000–$19,999	21 (41%)	10 (24%)	4 (27%)	
> $20,000	12 (24%)	17 (40%)	4 (27%)	
(Missing)	1	1	0	
Employment				**0.027**
Employed	9 (17%)	8 (19%)	3 (20%)	
Self-employed	9 (17%)	11 (26%)	9 (60%)	
Not in labor force	31 (60%)	20 (47%)	2 (13%)	
Unemployed	3 (5.8%)	4 (9.3%)	1 (6.7%)	
Insurance				0.9
Insured	44 (86%)	37 (86%)	12 (80%)	
Uninsured	7 (14%)	6 (14%)	3 (20%)	
(Missing)	1	0	0	

Median (IQR); *n* (%). Kruskal–Wallis rank sum test; Pearson’s Chi-squared test; Fisher’s exact test

*Breast cancer screening behaviors* In addition to mammogram status and compliance with screening guidelines, the results show more precise information about the length of time since the last reported mammogram (Table 2). For those who are compliant with screening guidelines, based on the time of the survey, 58% have had a screening in the last 12 months and 42% have had a mammogram over 1 year ago but less than 2 years. For those who have had a mammogram, but are currently not compliant, 47% of respondents have had a mammogram more than 2 years ago but less than 3 years ago, which means that 53% of respondents have not had a mammogram in the last 3 years. Both groups of women who have been screened report that the main reason why they did their most recent mammogram was because it was part of a routine exam (81% for ever/compliant and 84% for ever/non-compliant). For those who have never been screened almost half (47%) said that they have not been screened because they perceive that a mammogram is too painful, unpleasant, or embarrassing. Answers varied widely to the questions about when women are supposed to start having mammograms and how often women over the age of 50 with no personal or family history of breast cancer should do mammograms.

**Table 2:** Self-reported behaviors and knowledge regarding breast cancer screening by mammogram screening status (*n* = 110)

	Compliant (*n* = 52)	Ever had test but not compliant (*n* = 43)	Never had test (*n* = 15)	*p* value
Have you ever had a mammogram?				
Yes	52 (100%)	43 (100%)	0 (0%)	
No	0 (0%)	0 (0%)	15 (100%)	
Most recent mammogram				
Less than 12 months ago	30 (58%)	0 (0%)		
1 to < 2 years	22 (42%)	0 (0%)		
2 to < 3 years	0 (0%)	20 (47%)		
3 to < 5 years	0 (0%)	14 (33%)		
5 to < 10 years	0 (0%)	7 (16%)		
10 years or more	0 (0%)	2 (4.7%)		
What was the main reason you had this mammogram done?				
Part of a routine exam	42 (81%)	36 (84%)		
Family history of breast cancer	2 (3.8%)	2 (4.7%)		
My healthcare provider told me I was high-risk	3 (5.8%)	1 (2.3%)		
Other	3 (5.8%)	1 (2.3%)		
Because of a breast problem	1 (1.9%)	2 (4.7%)		
I requested it	1 (1.9%)	1 (2.3%)		
Reasons respondents selected for not having had a mammogram				
Your doctor did not recommend it			4 (27%)	
The test is too painful, unpleasant, or embarrassing			7 (47%)	
Too expensive, no insurance, cost			2 (13%)	
Age; thought I was too young to have test			2 (13%)	
I do not have any problems			2 (13%)	
Breast cancer screening not important			3 (20%)	
At what age are women supposed to start having mammograms? Is it…				0.4
0–29 years	11 (21%)	8 (19%)	1 (6.7%)	
30–39 years	21 (40%)	18 (42%)	4 (27%)	
40–49 years	10 (19%)	11 (26%)	4 (27%)	
50–59 years	10 (19%)	6 (14%)	6 (40%)	
How often should women 50 years and older with no personal or family history of breast cancer do a mammogram?				0.2
Every year	24 (46%)	10 (23%)	9 (60%)	
Every 2 years	17 (33%)	18 (42%)	2 (13%)	
Every 3 years	6 (12%)	9 (21%)	2 (13%)	
Every 6 months	3 (5.8%)	3 (7.0%)	2 (13%)	
Every 5 years	2 (3.8%)	2 (4.7%)	0 (0%)	
Every 4 years	0 (0%)	1 (2.3%)	0 (0%)	

*n* (%); Fisher’s exact test

*Health status and access to medical care* Analysis of health status measures did not reveal statistically significant differences among the breast cancer screening status groups. However, four measures of access to medical care showed distinct associations for screening (Table 3). The groups varied by the percentage who reported having a regular health care provider (81% ever/compliant, 65% ever/non-compliant, and 47% never; *p*-value = 0.022). The percentages of respondents who see health care providers when they are not sick, but to do check-up visits also vary (61% ever/compliant, 37% ever/non-compliant, and 20% never; *p*-value 0.007). The median (IQR) number of visits to a healthcare provider over the last 12 months for any reason varied significantly (*p*-value < 0.001): the ever/compliant group reported 3 (2,4) visits compared to 2 (1,4) visits for the ever/non-compliant and 0 (0,1) visits for the never screened groups. Similarly, when asked how many visits respondents have made to an IHS clinic in the last 3 years, 71% of the ever/compliant group and 74% of the ever/non-compliant group reported having made three or more visits compared to 27% of the never screened group (*p*-value 0.02). While non-significant, there is a pattern worth noting about how respondents perceived their relationships with their providers. When participants were asked if their health care providers treated them with respect, the majority of each group agreed or agreed strongly (79% ever/compliant, 78% ever/non-compliant, and 94% never), but there are differences in the patterns of which groups disagreed (16% ever/compliant, 4.8% ever/non-compliant, and 6.7% never).

**Table 3:** Health status and access to medical care by mammogram screening status (*n* = 110)

	Compliant, (*n* = 52)	Ever had test but not compliant (*n* = 43)	Never had test (*n* = 15)	*p* value
Self-rated Health				0.1
Fair/poor	10 (19%)	5 (12%)	4 (27%)	
Good	22 (42%)	22 (51%)	10 (67%)	
Very good/excellent	20 (38%)	16 (37%)	1 (6.7%)	
How often do you exercise?				0.3
Daily or more than three times per week	15 (29%)	11 (26%)	8 (53%)	
One to three times per week	17 (33%)	18 (42%)	3 (20%)	
You do not exercise regularly	20 (38%)	14 (33%)	4 (27%)	
Personal cancer history (yes)	6 (12%)	3 (7.0%)	1 (6.7%)	0.8
Family cancer history (yes)	36 (69%)	32 (74%)	9 (60%)	0.6
Do you have a health care provider that you go to regularly? (yes)	42 (81%)	28 (65%)	7 (47%)	**0.022**
Where do you usually go for health care?				0.089
Doctor or other provider in a private clinic	3 (5.8%)	1 (2.3%)	0 (0%)	
Hospital outpatient clinic	1 (1.9%)	1 (2.3%)	3 (20%)	
I do NOT have a usual place for health care	1 (1.9%)	1 (2.3%)	1 (6.7%)	
IHS/tribal clinic or hospital	47 (90%)	40 (93%)	11 (73%)	
Do you ever go to see a health care provider when you are not sick or having any problems, just to get a check-up? (yes)				**0.007**
No	20 (39%)	27 (63%)	12 (80%)	
Yes	31 (61%)	16 (37%)	3 (20%)	
(Missing)	1	0	0	
In the past 12 months, how many times did you visit a health care provider for any reason?	3 (2, 4)	2 (1, 4)	0 (0, 1)	**< 0.001**
How many times have you been to an Indian Health Services clinic in the past three years?				**0.02**
Never	4 (7.7%)	2 (4.7%)	3 (20%)	
One time	3 (5.8%)	4 (9.3%)	4 (27%)	
Two times	8 (15%)	5 (12%)	4 (27%)	
Three times or more	37 (71%)	32 (74%)	4 (27%)	
The hospital or clinic I usually go to provides me with good health care overall. Do you…				0.8
Strongly agree	4 (7.7%)	2 (4.7%)	1 (6.7%)	
Agree	33 (63%)	30 (70%)	10 (67%)	
Neither agree nor disagree	8 (15%)	5 (12%)	1 (6.7%)	
Disagree	5 (9.6%)	6 (14%)	2 (13%)	
Strongly disagree	2 (3.8%)	0 (0%)	1 (6.7%)	
The health care providers I usually see treat me with dignity and respect. Do you…				0.14
Strongly agree	12 (24%)	6 (14%)	4 (27%)	
Agree	28 (55%)	27 (64%)	10 (67%)	
Neither agree nor disagree	3 (5.9%)	7 (17%)	0 (0%)	
Disagree	7 (14%)	2 (4.8%)	0 (0%)	
Strongly disagree	1 (2.0%)	0 (0%)	1 (6.7%)	
(Missing)	1	1	0	
I feel comfortable talking to health care providers when I have a health problem. Do you…				0.5
Strongly agree	14 (27%)	11 (26%)	2 (13%)	
Agree	32 (62%)	26 (60%)	13 (87%)	
Neither agree nor disagree	3 (5.8%)	2 (4.7%)	0 (0%)	
Disagree	1 (1.9%)	4 (9.3%)	0 (0%)	
Strongly disagree	2 (3.8%)	0 (0%)	0 (0%)	
Have you ever had a test to check for cervical cancer? (yes)	44 (85%)	37 (86%)	12 (80%)	0.9

*n* (%); Median (IQR). Fisher’s exact test; Pearson’s Chi-squared test; Kruskal–Wallis rank sum test

*Breast cancer risk knowledge* Women’s knowledge of breast cancer risks was measured by assessing correct knowledge of 21 factors (10 more medically reasonable, and 11 less medically reasonable) which increase a woman’s chance of developing breast cancer (Table 4). There were two factors that were significantly different among the groups. Those who correctly answered that a family history of breast cancer increases a woman’s chance of breast cancer varied by breast cancer screening status (88% ever/compliant and ever/non-compliant compared to 60% never; *p*-value 0.032). In addition, correctly answering that having breast implants increases a woman’s chance of breast cancer varied by breast cancer screening status (73% ever/compliant, 86% ever/non-compliant, and 40% never; *p*-value 0.003).

**Table 4:** Knowledge about breast cancer risk factors by mammogram screening status (*n* = 110)

Which of the following things increase a woman’s chance of developing breast cancer? (yes)	Compliant (*n* = 52)	Ever had test but not compliant (*n* = 43)	Never had test (*n* = 15)	*p* value
**Getting older**	36 (69%)	25 (58%)	10 (67%)	0.5
**Giving birth to 1st child after age 30**	11 (21%)	9 (21%)	4 (27%)	> 0.9
**Using birth control pills**	28 (54%)	23 (53%)	9 (60%)	> 0.9
**Having breast implants**	38 (73%)	37 (86%)	6 (40%)	**0.003**
**Starting menstruation before age 12**	16 (31%)	8 (19%)	4 (27%)	0.4
**Family history of breast cancer**	46 (88%)	38 (88%)	9 (60%)	**0.032**
**Drinking excessive alcohol**	33 (63%)	24 (56%)	7 (47%)	0.5
**Being overweight or obese**	28 (54%)	23 (53%)	7 (47%)	0.9
**Not being physically active**	26 (50%)	25 (58%)	9 (60%)	0.7
**Not having children**	15 (29%)	9 (21%)	4 (27%)	0.7
*A diet low in fruits and vegetables*	25 (48%)	17 (40%)	8 (53%)	*0.6*
*A diet low in fiber*	27 (52%)	21 (49%)	10 (67%)	0.5
*A diet high in fat*	43 (83%)	34 (79%)	12 (80%)	> 0.9
*A diet high in processed meats*	39 (75%)	30 (70%)	12 (80%)	0.8
*Smoking*	43 (83%)	39 (91%)	10 (67%)	0.1
*Receiving hits or bruises to the breast*	24 (46%)	28 (65%)	5 (33%)	0.056
*Excessive fondling of the breast*	11 (21%)	11 (26%)	3 (20%)	0.9
*Being exposed to medical X-rays*	31 (60%)	28 (65%)	8 (53%)	0.7
*Using preservatives in food*	24 (46%)	17 (40%)	6 (40%)	0.8
*Having many sexual partners*	22 (42%)	16 (37%)	4 (27%)	0.5
*Breast feeding*	14 (27%)	6 (14%)	1 (6.7%)	0.2

*n* (%). Pearson’s Chi-squared test; Fisher’s exact test

Bold text—more medically reasonable risk factors; Italics—less medically reasonable risk factors

While there are not statistically significantly different areas of breast cancer risk knowledge, there are response patterns worth noting. A majority of respondents from all breast cancer screening statuses did not correctly identify the following more medically reasonable risk factors: not having children, giving birth to first child after the age of 30, and starting menstruation before the age of 12. More than one third of respondents across all breast cancer screening categories did not correctly identify the following more medically reasonable risk factors: using birth control pills, drinking excessive alcohol, being overweight or obese, and not being physically active. In addition, women across all three breast cancer screening groups incorrectly identified many of the less medically reasonable factors as contributors to a woman’s change of developing breast cancer.

*Attitudes and beliefs about cancer* Among the items in the measures of attitudes and beliefs about cancer in general, three items had different associations with breast cancer screening status (Table 5). First, a greater percentage of those reporting having ever been screened (compliant and non-compliant) reported that they agreed that they were very likely to get cancer in their lifetime (56% ever/compliant, 44% ever/non-compliant, and 20% never; *p*-value 0.047). Second, a greater percentage of those reporting having ever been screened (compliant and non-compliant) reported that they agreed that it seems like almost everything causes cancer (31% ever/compliant, 40% ever/non-compliant, and 0% never; *p*-value 0.007). Finally, those who had reported screening were more likely to report having discussed their personal risk of cancer with a provider: 56% ever/compliant, 37% ever/non-compliant, and 20% never (*p*-value 0.027). Answers vary across many items, but no other associations were statistically significant.

**Table 5:** Attitudes and beliefs toward cancer in general by mammogram screening status (*n* = 110)

	Compliant (*n* = 52)	Ever had test but not compliant (*n* = 43)	Never had test (*n* = 15)	*p* value
Agreed that				
If cancer is found early, it can be cured	48 (92%)	38 (88%)	15 (100%)	0.5
I think I would rather not know if I had cancer	6 (12%)	3 (7.0%)	3 (20%)	0.3
At my age I do not need to worry about cancer	9 (17%)	6 (14%)	6 (40%)	0.1
I would undergo cancer treatment that is unpleasant or painful if it would improve my chances of living longer	46 (88%)	41 (95%)	15 (100%)	0.3
I would be afraid to tell my spouse that I have cancer because I think it would affect our relationship	6 (12%)	6 (14%)	1 (6.7%)	0.8
There is not much that I can do to prevent getting cancer	21 (40%)	13 (30%)	8 (53%)	0.3
I am very likely to get cancer in my lifetime	29 (56%)	19 (44%)	3 (20%)	**0.047**
Benefits of getting a test to screen for cancer are greater than any inconvenience	40 (77%)	34 (79%)	12 (80%)	> 0.9
My family and friends would support me in doing a test to screen for cancer	51 (98%)	38 (88%)	15 (100%)	0.11
With regards to doing a test to screen for cancer, I want to do what my family and friends think I should do	32 (62%)	32 (74%)	10 (67%)	0.4
There is not much you can do to lower your chances of getting cancer	18 (35%)	9 (21%)	6 (40%)	0.2
There are so many different recommendations about preventing cancer that it is hard to know which ones to follow	41 (79%)	31 (72%)	14 (93%)	0.2
Cancer develops over a period of several years	37 (71%)	31 (72%)	12 (80%)	0.9
There are ways to slow down or disrupt the development of cancer	41 (79%)	34 (79%)	14 (93%)	0.5
Cancer is most often caused by a person’s behavior or lifestyle	20 (38%)	19 (44%)	5 (33%)	0.7
It seems like almost everything causes cancer	16 (31%)	17 (40%)	0 (0%)	**0.007**
You are reluctant to get checked for cancer because you fear you may have it	20 (38%)	15 (35%)	5 (33%)	> 0.9
People with cancer would have pain or other symptoms prior to being diagnosed	38 (73%)	29 (67%)	14 (93%)	0.14
A person can have cancer without symptoms	41 (80%)	36 (84%)	11 (73%)	0.7
Yes				
Family or friends have suggested to do a test to screen for cancer	15 (29%)	16 (38%)	3 (20%)	0.4
Would do a test to screen for cancer if encouraged by health care provider	50 (96%)	39 (93%)	14 (93%)	0.6
Would be able to request a test to screen for cancer from your health care provider	42 (81%)	39 (91%)	13 (87%)	0.4
Has discussed personal risk for cancer with friends or relatives	22 (42%)	15 (35%)	4 (27%)	0.5
Has discussed personal risk cancer with provider	29 (56%)	16 (37%)	3 (20%)	**0.027**
How likely are you to do a test to screen for cancer within the next 12 months?				0.5
Not at all likely	11 (21%)	5 (12%)	3 (20%)	
A little likely	5 (9.6%)	6 (14%)	4 (27%)	
Somewhat likely	16 (31%)	10 (23%)	2 (13%)	
Likely	14 (27%)	12 (28%)	3 (20%)	
Very likely	6 (12%)	10 (23%)	3 (20%)	
Compared to other people your age, how likely do you think it is that you could get cancer? Are you…				0.5
Much less likely	6 (12%)	2 (4.7%)	2 (13%)	
Less likely	8 (16%)	8 (19%)	3 (20%)	
No difference	12 (24%)	14 (33%)	7 (47%)	
More likely	20 (41%)	15 (35%)	2 (13%)	
Much more likely	3 (6.1%)	4 (9.3%)	1 (6.7%)	
(Missing)	3	0	0	
Some people say that they either get cancer or they do not get it. They believe that there is nothing that can be done to prevent getting cancer. Do you…				0.5
Strongly disagree	11 (22%)	10 (23%)	1 (6.7%)	
Disagree	17 (33%)	14 (33%)	2 (13%)	
Neither agree nor disagree	4 (7.8%)	3 (7.0%)	3 (20%)	
Agree	13 (25%)	11 (26%)	6 (40%)	
Strongly agree	6 (12%)	5 (12%)	3 (20%)	
(Missing)	1	0	0	
Some people say that a person gets cancer as punishment for something they have done wrong. Do you…				0.4
Strongly disagree	23 (45%)	21 (49%)	4 (27%)	
Disagree	21 (41%)	19 (44%)	7 (47%)	
Neither agree nor disagree	4 (7.8%)	2 (4.7%)	2 (13%)	
Agree	3 (5.9%)	1 (2.3%)	1 (6.7%)	
Strongly agree	0 (0%)	0 (0%)	1 (6.7%)	
(Missing)	1	0	0	

*n* (%).Fisher’s exact test; Pearson’s Chi-squared test

Although there are a range of differences in both knowledge and beliefs among these groups, women across all three screening statuses reported that they would get screened if a health care provider encouraged them to (96% ever screened/compliant, 93% ever screened/not compliant, and 93% never screened). One additional standout difference is that of the women who got screening, most women disagreed or strongly disagreed that with the statement “some people say that they either get cancer or they do not get it. They believe there is nothing that can be done to prevent getting cancer” (55% disagreed/strongly disagreed of ever/compliant and 56% disagreed/strongly disagreed of ever/non-compliant), but of the women who have never been screened most women agreed or strongly agreed with the statement (60% agreed/strongly agreed of never).

Finally, women reported *preferences for strategies to improve cancer screening* (Table 6). For women who have been screened (whether compliant or non-compliant), at least 75% reported a preference for 13 of the 15 strategies suggested to improve cancer screening. The two strategies which were not as highly preferred were the same for both groups [offering childcare services (69% ever/compliant and 72% ever/non-compliant) and using social media such as Facebook, YouTube, Twitter (46% ever/compliant and 53% ever/non-compliant)]. However, for women who have never been screened, only 6 of the 15 strategies suggested garnered the preference of at least 75% of women. The four highest preferred strategies (all tied at 87%) for those who have never been screened were: (1) one-on-one education, (2) having community health representatives (CHRs) or patient navigators help obtain screening, (3) reminders such as postcards, emails, or phone messages, and (4) having flexible clinic hours. The two least preferred strategies were home visits for education and using social media such as Facebook, YouTube, Twitter (both tied at 53%).

**Table 6:** Preference for strategies to improve cancer screening by mammogram screening status (*n* = 110)

Selected the following	Compliant (*n* = 52)	Ever had test but not compliant (*n* = 43)	Never had test (*n* = 15)	*p* value
Printed materials such as letters, brochures, and newsletters	49 (94%)	39 (91%)	12 (80%)	0.2
One-on-one education	48 (92%)	40 (93%)	13 (87%)	0.8
Having community health representatives (CHRs) or patient navigators help obtain screening	48 (92%)	36 (84%)	13 (87%)	0.4
Reminders such as postcards, emails, or phone messages	47 (90%)	41 (95%)	13 (87%)	0.3
Videos in the clinic waiting room	45 (87%)	40 (93%)	11 (73%)	0.14
Having flexible clinic hours	45 (87%)	35 (81%)	13 (87%)	0.8
Offering transportation to the clinic	44 (85%)	35 (81%)	11 (73%)	0.6
Group education	44 (85%)	33 (77%)	9 (60%)	0.14
Offering translation or interpretation services at the clinic	43 (83%)	39 (91%)	11 (73%)	0.3
Offering screening through non-clinical settings such as mailing for colorectal cancer screening	42 (81%)	33 (77%)	11 (73%)	0.8
Reducing co-payments for testing	42 (81%)	39 (91%)	10 (67%)	0.086
Public service announcements (PSAs) on the radio	41 (79%)	36 (84%)	12 (80%)	0.8
Home visits for education	39 (75%)	35 (81%)	8 (53%)	0.1
Offering child-care services	36 (69%)	31 (72%)	10 (67%)	0.9
Using social media such as Facebook, YouTube, twitter	24 (46%)	23 (53%)	8 (53%)	0.7

*n* (%). Fisher’s exact test; Pearson’s Chi-squared test

## Discussion

This descriptive study of Zuni women’s breast cancer and more general cancer screening attitudes, beliefs, and behaviors and the association with breast cancer screening compliance guidelines shows strengths and areas where interventions may be able to boost breast cancer screening. Zuni women who responded to the survey have a relatively high rate of ever having had a breast cancer screening (86%), and higher percentages of Zuni women in this study report being compliant with screenings (47%) compared to the 2019 IHS numbers for all AI/AN women (42%) and for the Albuquerque Area (38.7%) [4]. Of course, all of these numbers fall short of the Health People 2020 goal of 81.0%, and all of these numbers mean that there is the potential to improve screening numbers which will reduce the burden of breast cancer for Zuni women and their families. Of the women who had been screened and were not compliant, 47% were within 12 months of compliance. As this survey was administered during the pandemic, some of these delays may be linked to pandemic related delays in elective health care. However, as over half reported having had their screening over 3 years prior, there is evidence that interventions should be tailored both for women who have had a screening but are not up-to-date with their screenings and for women who have never had a screening. Having increased clinic hours, which was a highly preferred strategy for improving cancer screening, would create more opportunities for community members to interact with a healthcare provider on a regular basis.

Healthcare providers matter. Both groups of women who report having been screened assert that the main reason why they did their most recent mammogram was because it was part of a routine exam (81% for ever/compliant and 84% for ever/non-compliant), and women across all three screening statuses reported that they would get screened if a health care provider encouraged them to (96% ever screened/compliant, 93% ever screened/not compliant, and 93% never screened). However, only 56% of women who have been screened and are compliant report having discussed personal risk of cancer more generally with their provider which is a higher percentage than the other groups. This takeaway is reinforced when looking at the fact that a greater percentage of those who were compliant reported having a regular provider than those who had been screened but were non-compliant and those who had never been screened. Regular visits, both for check-ups and specific concerns, also provide connection to healthcare which may be a good opportunity to promote breast cancer screening.

More good news is that the majority of respondents agreed that their healthcare providers treated them with respect, but there is still room for improvement, especially as the group that has the highest reported interactions with providers—those who have ever been screened and are compliant—report the highest percentage of disagreement that their providers treat them with respect. With trust and culturally tailored interventions, providers have the potential to improve breast cancer screening for Zuni women.

This study provides a foundation for how to start building culturally tailored interventions. As the most common response for never being screened is that women perceive a mammogram as too painful, unpleasant, or embarrassing, there is an opportunity to educate women about the test and to support them through the vulnerable moments that may come along with mammography. One pathway to help motivate women to be screened is to help them understand the benefits of early screening. Women from all groups had gaps in knowledge about breast cancer and cancer risk factors. Most women did know that family history of breast cancer increased their own risk. This is a great foundation. While most women also knew that breast implants increased a woman’s chance of cancer, this may be less impactful as this is relevant to a smaller subset.

An evidence-based intervention building on these results would clearly communicate when women should start having mammograms and how often. It would review how a woman’s personal health history including the age of menarche, the age at first childbirth, or not having children all contribute to breast cancer risk. It would also review health behaviors including how using birth control, alcohol consumption patterns, weight, and physical activity all contribute to breast cancer risk. Building on areas where there are gaps in knowledge may more quickly build capacity for higher rates of breast cancer screening.

Finally, any intervention should support women’s belief in themselves to impact their health. While there are many structural and hereditary factors that influence one’s risk of breast cancer, early detection can reduce the overall burden by having less invasive and more effective treatment. The result that 37% and 38% of women who had been screened and were compliant or non-complaint, respectively, but that 60% of women who had not been screened agreed or strongly agreed that people either get cancer or they do not and that there is nothing which can be done to prevent it, shows that many women do not believe that they can have an influence over breast cancer’s impact in their lives. By building women’s knowledge of breast cancer and breast cancer screening, screening’s role in reducing the burden of breast cancer, interventions have the potential to impact breast cancer screening beliefs and behavior for Zuni women.

Finally, this survey also establishes a foundation for providers to implement effective cancer screening prevention programs. For this group of women, social media interventions are the least preferred. For the group of women who have never been screened, they would prefer one-on-one outreach, assistance with obtaining screening, multiple reminders, and flexible clinic hours. That said, all groups of women strongly preferred many different intervention program suggestions. By developing programs based on the knowledge and belief findings from this study and combining those with preferred intervention programs, providers and public health educators would have a solid foundation of an evidence-based intervention to improve breast cancer screening for Zuni women.

## Data Availability

Enquiries about data availability should be directed to the authors.
